# Protective immunity against toxoplasmosis in mice induced by single-dose immunization with rSAG1/2 protein released from poly(lactide-co-glycolide) microparticles

**DOI:** 10.1051/parasite/2017004

**Published:** 2017-02-01

**Authors:** Shu-Chun Chuang, Yao-Chi Chung, Chung-Da Yang

**Affiliations:** 1 Orthopaedic Research Center and Department of Physiology, College of Medicine, Kaohsiung Medical University No. 100, Shih-Chuan 1st Road Kaohsiung 807 Taiwan; 2 Graduate Institute of Animal Vaccine Technology, College of Veterinary Medicine, National Pingtung University of Science and Technology No. 1, Shuefu Road Neipu, Pingtung 912 Taiwan

**Keywords:** *Toxoplasma gondii*, Single-dose vaccine, rSAG1/2, Synthetic adjuvant, Poly(lactide-co-glycolide), Microparticles

## Abstract

Triphasic sustained release of tachyzoite chimeric protein, rSAG1/2, from poly(lactide-co-glycolide) (PLG)-encapsulated rSAG1/2 (PLG-rSAG1/2) microparticles (MPs) is a promising characteristic for developing a single-dose vaccine against *Toxoplasma gondii* in domestic animals. In the present study, we aimed to evaluate whether single immunization with PLG-rSAG1/2 MPs in BALB/c mice would achieve effective immunity and protection against *T. gondii*. Peritoneal immunization of mice with a single dose of PLG-rSAG1/2 MPs enhanced serum IgG titers and lymphocyte proliferation in a triphasic model over a long 12-week period. In addition, 12 weeks after immunization, significant production of IFN-γ was also monitored in mice vaccinated with one dose of PLG-rSAG1/2 MPs. More importantly, the immunity induced by one dose of PLG-rSAG1/2 MPs protected 70% of mice (14/20) against a lethal subcutaneous challenge of 1 × 10^4^ live tachyzoites of *T. gondii* (RH strain). In conclusion, a single dose of PLG-rSAG1/2 MPs capable of sustaining triphasic release of rSAG1/2 protein induces long-lasting triphasic immunity against *T. gondii* in mice. Our data indicate the feasibility of PLG-rSAG1/2 MPs to be developed as a single-dose vaccine against *T. gondii* for potential use in domestic animals.

## Abstract (fr)

La libération prolongée triphasique de la protéine chimère du tachyzoïte rSAG1/2, à partir de microparticules (MPs) de rSAG1/2 encapsulées par le poly (lactide-co-glycolide) (PLG-rSAG1/2), est une caractéristique prometteuse pour le développement d’un vaccin en dose unique contre *Toxoplasma gondii* chez les animaux domestiques. Dans la présente étude, nous avons cherché à évaluer si une seule immunisation avec des MPs PLG-rSAG1/2 chez les souris BALB/c permettrait d’obtenir une immunité efficace et une protection contre *T. gondii*. L’immunisation péritonéale de souris avec une dose unique de MPs PLG-rSAG1/2 a amélioré les titres d’IgG sérique et la prolifération lymphocytaire dans un modèle triphasique sur une période de 12 semaines. De plus, douze semaines après immunisation, une production significative d’IFN-γ a également été remarquée chez des souris vaccinées avec une dose de MPs PLG-rSAG1/2. Plus important encore, l’immunité induite par une dose de MPs PLG-rSAG1/2 a protégé 70 % (14/20) des souris contre une injection létale sous-cutanée de 1 × 10^4^ tachyzoïtes vivants de *T. gondii* (souche RH). En conclusion, une dose unique de MPs PLG-rSAG1/2, capable de maintenir la libération triphasique de la protéine rSAG1/2, induit une immunité triphasique de longue durée contre *T. gondii* chez la souris. Nos données indiquent la faisabilité des MPs PLG-rSAG1/2, à développer comme un vaccin à dose unique contre *T. gondii* pour une utilisation potentielle chez les animaux domestiques.

## Introduction

*Toxoplasma gondii* is an intracellular protozoan parasite that causes serious toxoplasmosis in most endothermic animals, including humans and domestic animals [11, 23]. Toxoplasmosis usually generates severe abortion and neonatal loss in domestic animals, thereby leading to dramatic economic losses [8, 25]. Toxoplasmosis during pregnancy may induce serious fetal congenital intellectual disability, blindness, and hydrocephaly [7, 30]. In addition, toxoplasmosis is also a major opportunistic infection in immunocompromised individuals, often resulting in lethal toxoplasmic encephalitis [6]. Although one attenuated vaccine has been used successfully to reduce abortions in sheep [2], it has a very short shelf-life and is unlikely to be used in humans [14]. Numerous recombinant vaccines derived from surface antigens, dense granule proteins, rhoptry proteins, and microneme proteins have produced only little to moderate protective efficacy against infections with a lethal challenge dose of different strains of *T. gondii* [33]. The lack of effective vaccines has become a major burden in controlling toxoplasmosis [19, 29].

Significant evidence obtained recently indicates that future investigations on the development of *Toxoplasma* vaccines have to include efficacious adjuvants that can improve vaccine immunogenicity for inducing the “appropriate” immunity against *T. gondii* in animals [14]. Microparticles (MPs) made from biodegradable and biocompatible polymers, such as poly(lactide-co-glycolide) (PLG), have been employed as safe and potent synthetic adjuvants to encapsulate antigens for producing controlled-release MP vaccines [10, 24]. Moreover, such antigen-controlled release is a particularly attractive characteristic of antigen-loaded PLG MPs for the development of single-dose vaccines without additional administration of booster doses [9, 12].

In our previous study, the chimeric tachyzoite surface antigen, rSAG1/2, was encapsulated with PLG polymer to prepare PLG-encapsulated rSAG1/2 (PLG-rSAG1/2) MPs [3]. In addition, the *in vitro* release of rSAG1/2 protein from PLG MPs suspended in PBS could be sustained for a 56-day period with three distinct phases consisting of an initial burst release, a very slow release, and a final rapid release [3]. Further protection analysis in mice demonstrated that two shots of PLG-rSAG1/2 MPs protected 83% of animals against a lethal subcutaneous challenge of *T. gondii* tachyzoites [3]. However, administration of multi-dose vaccines to achieve protective immunity is usually cost-ineffective, complex and its compliance is frequently difficult for use in complete vaccination of domestic animals [9]. Therefore, the *in vitro* triphasic sustained release of rSAG1/2 protein provides valuable potential, encouraging us to evaluate whether vaccination with a single dose of PLG-rSAG1/2 MPs could achieve *in vivo* protection against *T. gondii* in animals.

In the present study, to further the development of anti-*Toxoplasma* MP vaccine for domestic animals, we aimed to evaluate whether single immunization with PLG-rSAG1/2 MPs in BALB/c mice would achieve effective immunity and protection against *T. gondii*. Mice were intraperitoneally immunized with a single dose of PLG-rSAG1/2 MPs and their anti-*Toxoplasma* immune responses were examined and compared with those induced by one or two intraperitoneal shot(s) of the oil formulation, rSAG1/2 (Vet L-10) [3, 31]. In addition, a lethal subcutaneous challenge with *T. gondii* tachyzoites was performed to assess protective activities induced by single immunization with the MP vaccine.

## Materials and methods

### Ethics statement

Female BALB/c mice (6 ~ 8 weeks of age) were purchased from the National Laboratory Animal Center (NLAC), Taiwan. All mice were housed in high containment facilities and managed in compliance with the Animal Welfare Act. All procedures in animal experiments were reviewed and approved by The Institutional Animal Care and Use Committee (IACUC), National Pingtung University of Science and Technology (NPUST) and all possible efforts were made to minimize the suffering of the experimental mice.

### Parasite antigens and monoclonal antibody (mAb)

*T. gondii* tachyzoites (RH strain) were harvested, purified, and sonicated to prepare the tachyzoite sonicated antigen (TsoAg) as described previously [3, 4]. In addition, the rSAG1/2 protein used in the present study was prepared from the RH strain of *T. gondii* tachyzoites in our previous study [3]. Mouse mAbs TG-1 (isotype G, subclass 1, κ light chain) and TG-2 (isotype G, subclass 1, κ light chain), which are, respectively, specific to SAG1 (30 kDa) and SAG2 (22 kDa), were prepared by fusion, selection, and cloning as described previously [4].

### Preparation of PLG-rSAG1/2 MPs

The PLG-rSAG1/2 MPs used for the immunization experiments in the present study were prepared by using a water/oil/water double emulsion method described in our previous work [3]. The resulting PLG-rSAG1/2 MPs, 1.27 ~ 1.65 μm in diameter, showed 72 ~ 83% entrapment efficiency. Moreover, the *in vitro* cumulative release of rSAG1/2 from MPs in PBS gradually increased over the course of a 56-day period with three distinct phases. Within the first three days, an initial burst released approximately 32.4% of the total protein load. Afterwards, there was a very slow release for 48 days, followed by a rapid release during the last five days. Altogether, 88.5% of the total protein load was released from the MPs during the 56-day period.

### Animal experiments

In order to study whether PLG-rSAG1/2 MPs could work as an effective single-dose vaccine against *T. gondii*, five groups of 41 BALB/c mice each were intraperitoneally immunized once with PLG-rSAG1/2 MPs containing 10 μg of rSAG1/2 protein, 10 μg of rSAG1/2 formulated with Invitrogen Vet L-10 adjuvant (rSAG1/2 (Vet L-10)) [3], 10 μg of rSAG1/2 alone, blank PLG, or PBS. An additional 41 BALB/c mice intraperitoneally injected twice at a 14-day interval with rSAG1/2 (Vet L-10) were also studied to evaluate whether single immunization with PLG-rSAG1/2 MPs would be superior to two-time immunization with the Vet L-10 oil formulation.

Three mice per group were euthanized at weeks 0, 2, 4, 6, 8, 10, and 12 to collect sera and spleen lymphocytes for assaying anti-*Toxoplasma* immune responses. Twelve weeks after immunization, the remaining mice in all groups (20/group) were challenged with a subcutaneous injection of 1 × 10^4^ live tachyzoites of *T. gondii* (RH strain) suspended in 100 μL of PBS in order to verify whether the induced immunity could protect mice from tachyzoite infection. Mice were monitored daily for an additional 28 days [5] and the survival rate in each group was calculated as described previously [3, 4].

### Serum assay

Four weeks after immunization, the antigenic specificity of mouse sera was studied by Western blot and anti-*Toxoplasma* IgG titers of mouse sera collected every two weeks were further measured by ELISA as described previously [3, 4].

### Lymphocyte proliferation assay

Every two weeks, three mice per group were euthanized to isolate spleen lymphocytes under sterile conditions. Then, 1 × 10^5^ cells in 200 μL of RPMI-1640 culture medium (CM) were cultured in each well of 96-well culture plates and stimulated with 5 μg/mL of TsoAg (containing native SAG1 and SAG2) as described previously [3]. In addition, CM-treated cultures without TsoAg stimulation were conducted and used as controls. The TsoAg-induced lymphocyte proliferation was then monitored using the BrdU (5-bromo-2′-deoxyuridine) Colorimetric Cell Proliferation ELISA Kit (Roche) according to the manufacturer’s instructions. Finally, the stimulation index (SI = OD_450_ values from TsoAg-treated cultures/OD_450_ values from CM-treated control cultures) of each group was calculated as described previously [3, 4].

### Measurement of IFN-γ production by real-time PCR

Twelve weeks after immunization, spleen lymphocytes isolated from different groups of mice were seeded in triplicate in 6-well culture plates at 1 × 10^6^ cells per well in 2 mL of RPMI-1640 medium. Cells were cultured for 18 h and then stimulated with 5 μg/mL TsoAg or 1 μg/mL of Con A (Sigma), a T-cell mitogen, for an additional 6 h. The Con A-stimulated cells were used as positive controls. In addition, CM-treated cultures without stimulation were also prepared. After incubation, total cellular RNA was extracted to reversely transcribe into cDNA for measuring IFN-γ production. The induced IFN-γ mRNA was then measured by real-time PCR with specific IFN-γ primers (forward: 5′-TCA AGT GGC ATA GAT GTG GAA GAA-3′ and reverse: 5′-TGG CTC TGC AGG ATT TTC ATG-3′) [21] by using a SYBR Green system in a LightCycler instrument (Roche Applied Science). Thermocycling conditions consisted of an initial denaturation (95 °C for 10 min) followed by 40 cycles of 15 s denaturation at 95 °C, 2 s annealing at 60 °C, and 15 s extension at 72 °C. Fluorescence curves were analyzed with use of LightCycler software, version 3.0 (Roche Applied Science). For each sample, the amount of IFN-γ was determined by comparing with a standard curve and normalized by using β-actin (forward: 5′-AGA GGG AAA TCG TGC GTG AC-3′ and reverse: 5′-CAA TAG TGA TGA CCT GGC CGT-3′) as the internal reference [21]. Results were expressed as the fold change that was determined by dividing the quantity of specific IFN-γ mRNA from stimulated cells by the quantity of IFN-γ mRNA from the unstimulated cells. All samples were processed in triplicate.

### Statistical analysis

Serum IgG titers (log_10_) and SI values of different groups were statistically compared by using the Nested design and the means at different time points in each group were tested by least significant difference (LSD) multiple comparison. IFN-γ fold changes from different groups were statistically compared using one-way ANOVA. The survival rates of different groups were analyzed by the chi-square test. A *P* value of less than 0.05 was considered to be significant.

## Results

### Antigenic specificity elicited by single immunization with PLG-rSAG1/2 MPs

**Figure 1.**
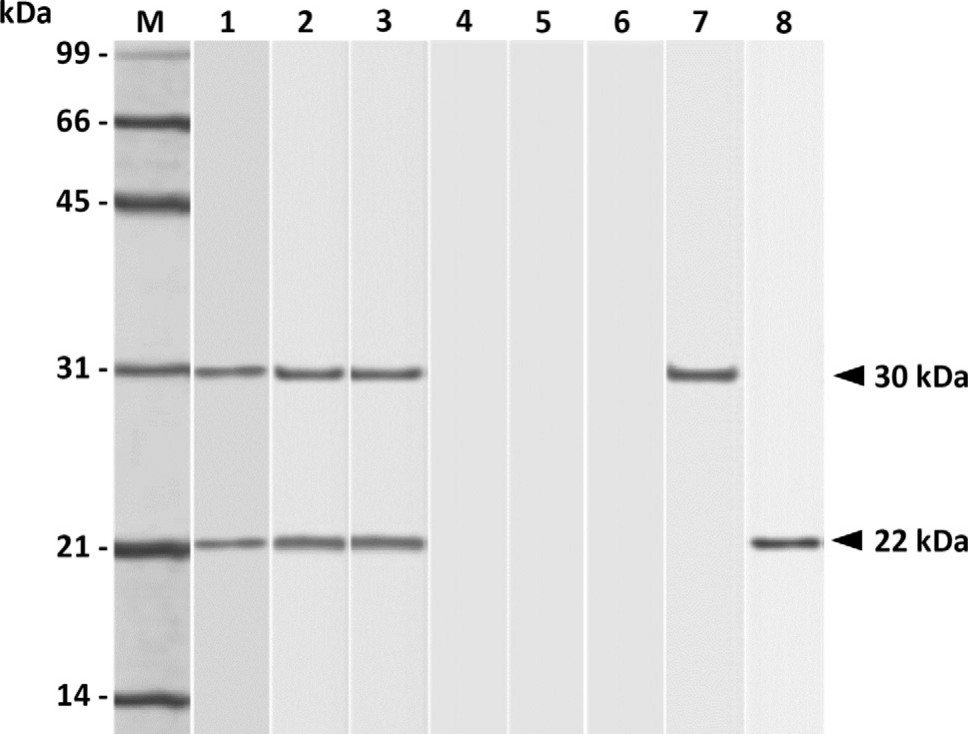
*Western blot analyses for antigenic specificity of immunized mouse sera*. Four weeks after immunization, TsoAg was probed with sera from mice immunized with one dose of PLG-rSAG1/2 MPs (lane 1), one dose of rSAG1/2 (Vet L-10) (lane 2), two doses of rSAG1/2 (Vet L-10) (lane 3), one dose of rSAG1/2 alone (lane 4), one dose of blank PLG (lane 5) or PBS (lane 6). In addition, the mouse mAbs TG-1 (lane 7) and TG-2 (lane 8) were also used as markers for SAG1 and SAG2, respectively. Standard protein markers (lane M) are shown on the left.

The antigenic specificity of mouse sera elicited four weeks after peritoneal immunization was studied by Western blot (Fig. 1). Results showed that one shot of PLG-rSAG1/2 MPs or oil formulation rSAG1/2 (Vet L-10) could elicit IgG antibodies to react to the native SAG1 (30 kDa) and SAG2 (22 kDa) in TsoAg (Fig. 1, lanes 1 and 2), which were also, respectively, recognized by the TG-1 and TG-2 mAbs (Fig. 1, lanes 7 and 8). In addition, mice immunized twice with oil formulation rSAG1/2 (Vet L-10) also induced the same IgG reaction (Fig. 1, lane 3). However, mice peritoneally immunized with rSAG1/2 alone, blank PLG, or PBS did not produce serum IgG antibodies in response to any proteins in TsoAg (Fig. 1, lanes 4–6). Thus, the rSAG1/2 protein formulated with adjuvants such as PLG polymer and Vet L-10 could elicit both anti-SAG1 and anti-SAG2 IgG antibodies in the mouse serum following one- or two-time immunization.

### IgG titers enhanced by single immunization with PLG-rSAG1/2 MPs

**Figure 2.**
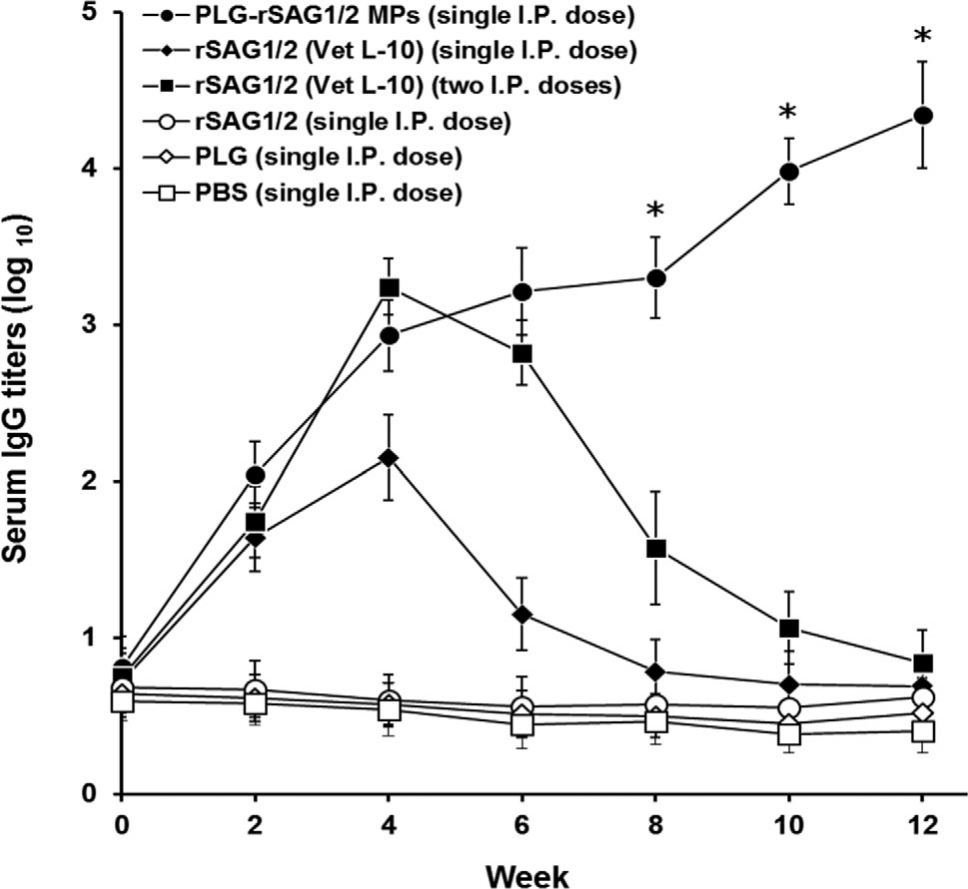
*Analyses for IgG titers of immunized mouse sera*. Groups of mice were peritoneally immunized with one dose of PLG-rSAG1/2 MPs (●), one dose of rSAG1/2 (Vet L-10) (◆), two doses of rSAG1/2 (Vet L-10) (■), one dose of rSAG1/2 alone (○), one dose of blank PLG (◇), or one dose of PBS (□). Every two weeks, mouse sera were collected from different groups and their anti-*Toxoplasma* IgG titers were determined by ELISA. Results are presented as the mean of log_10_ titers ± SD. All groups were analyzed by the Nest design and an asterisk (*) indicates *P <* 0.05 when comparing the PLG-rSAG1/2 (single I.P. dose) group to the rSAG1/2 (Vet L-10) (two I.P. doses) group.

Every two weeks, anti-*Toxoplasma* IgG titers of mouse sera were further determined by ELISA (Fig. 2). Before immunization (week 0), different groups of mice were found to show similarly low serum IgG titers. After single immunization with PLG-rSAG1/2 MPs, IgG titers initially increased from week 0 to week 4 (*P* < 0.05, LDS multiple comparison), then slowly and insignificantly increased from week 4 to week 8 (*P* > 0.05, LDS multiple comparison), and finally increased once again during the last four weeks (from week 8 to week 12) (*P* < 0.05, LDS multiple comparison). Thus, a triphasic profile of anti-*Toxoplasma* IgG titers could be elicited in mice by peritoneal vaccination with one dose of PLG-rSAG1/2 MPs. On the other hand, high IgG titers induced by two shots of rSAG1/2 (Vet L-10) during the first four weeks (*P* < 0.05, LDS multiple comparison) then gradually decreased to the levels before immunization from week 4 to week 12 (*P* < 0.05, LDS multiple comparison). In further comparison, one shot of PLG-rSAG1/2 MPs and two shots of rSAG1/2 (Vet L-10) induced similar IgG titers in the first six weeks (*P* > 0.05, Nested design). More importantly, at all indicated time points from week 6 to week 12, IgG titers elicited by one shot of PLG-rSAG1/2 MPs remained significantly higher than those induced by two shots of rSAG1/2 (Vet L-10) (*P* < 0.05, Nested design). Mice given one dose of rSAG1/2 (Vet L-10) via the intraperitoneal route showed a mild IgG induction in the first four weeks and then a gradual reduction starting from the 4th week. However, very low anti-*Toxoplasma* IgG titers were found in mice following single peritoneal immunization with soluble rSAG1/2 alone, blank PLG, or PBS. Therefore, peritoneal immunization with one dose of rSAG1/2-loaded PLG MPs in mice could induce and prolong anti-*Toxoplasma* IgG titers in a triphasic model over a 12-week period.

### Lymphocyte proliferation induced by single immunization with PLG-rSAG1/2 MPs

**Figure 3.**
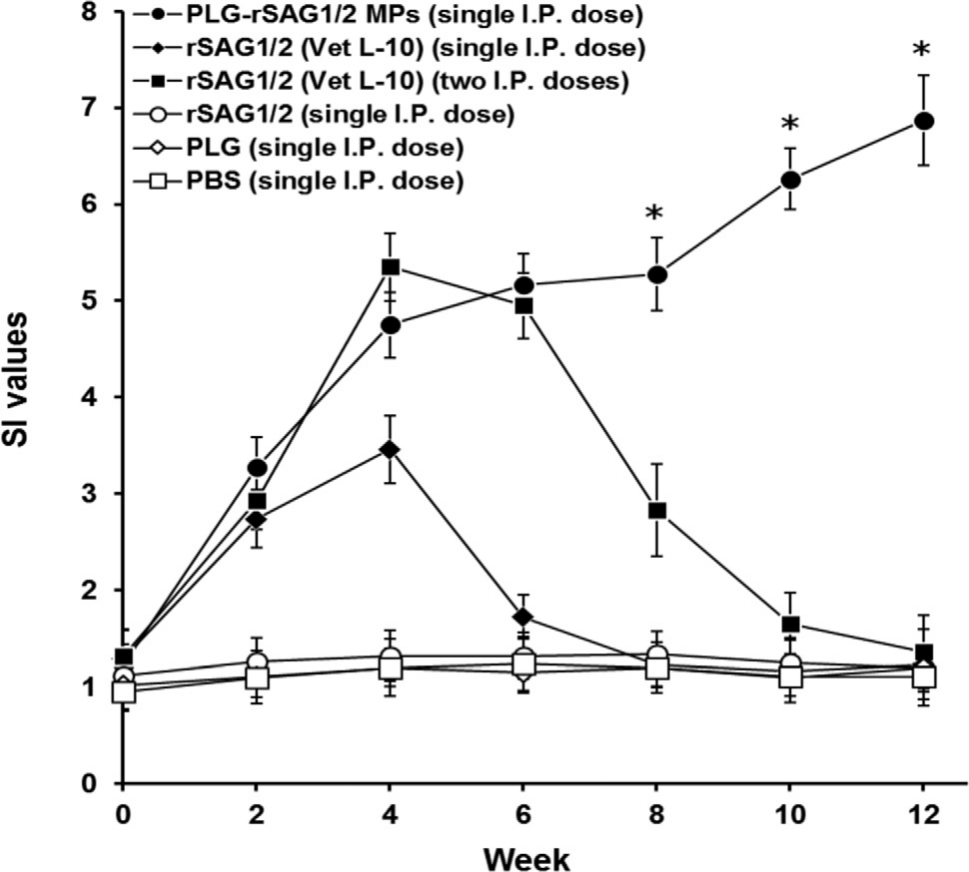
*Analyses for lymphocyte proliferation of immunized mice*. Groups of mice were peritoneally immunized with one dose of PLG-rSAG1/2 MPs (●), one dose of rSAG1/2 (Vet L-10) (◆), two doses of rSAG1/2 (Vet L-10) (■), one dose of rSAG1/2 alone (○), one dose of blank PLG (◇), or one dose of PBS (□). Every two weeks, the TsoAg-induced proliferative responses of spleen lymphocytes in each group were calculated by the BrdU (5-bromo-2′-deoxyuridine) Colorimetric Cell Proliferation ELISA kit (Roche). Results are expressed as mean ± SD. All groups were analyzed by the Nest design and an asterisk (*) indicates *P <* 0.05 when comparing the PLG-rSAG1/2 (single I.P. dose) group to the rSAG1/2 (Vet L-10) (two I.P. doses) group.

Spleen lymphocytes were prepared from different mouse groups every two weeks and their proliferative responses specific to TsoAg were studied and expressed as SI values (Fig. 3). During the 12-week study, mice intraperitoneally immunized with one dose of PLG-rSAG1/2 MPs produced a triphasic induction in SI values, which consisted of an initial increase in the first four weeks (*P* < 0.05, LDS multiple comparison), a slow and insignificant increase during the following four weeks (*P* > 0.05, LDS multiple comparison) and a second increase during the last four weeks (*P* < 0.05, LDS multiple comparison). Although an initial induction in SI values was also elicited by two peritoneal shots of rSAG1/2 (Vet L-10) in first four weeks (*P* < 0.05, LDS multiple comparison), the induced SI values gradually decreased from week 4 to week 12 (*P* < 0.05, LDS multiple comparison). Furthermore, in the first six weeks, similar high SI values were observed in mice immunized with one shot of PLG-rSAG1/2 MPs or two shots of rSAG1/2 (Vet L-10) (*P* > 0.05, Nested design). However, from week 6 to week 12, one dose of PLG-rSAG1/2 MPs maintained significantly higher SI values than two doses of rSAG1/2 (Vet L-10) (*P* < 0.05, Nested design). Mild SI values induced by single peritoneal immunization with rSAG1/2 (Vet L-10) in the first four weeks gradually decreased to the levels prior to immunization starting from week 4. However, single peritoneal administration of soluble rSAG1/2 alone, blank PLG, or PBS in mice resulted in little, if any, lymphocyte proliferation. Thus, peritoneal immunization with a single dose of rSAG1/2 protein encapsulated into PLG MPs could enhance and maintain anti-*Toxoplasma* lymphocyte proliferation in a triphasic 12-week profile.

### IFN-γ production elicited by single immunization with PLG-rSAG1/2 MPs

Before challenge (the 12th week), the TsoAg- or Con A-stimulated spleen lymphocyte cultures derived from different mouse groups were harvested to assess their fold changes of IFN-γ production by real-time PCR (Table 1). Upon TsoAg stimulation, high IFN-γ fold changes in spleen lymphocytes were only found in mice peritoneally immunized with a single dose of PLG-rSAG1/2 MPs. However, spleen lymphocytes from the other five groups of mice showed similar low fold changes (*P* > 0.05, ANOVA). Thus, before challenge, single peritoneal immunization with PLG-rSAG1/2 MPs generated significantly higher levels of IFN-γ in mice than peritoneal immunization with one or two shot(s) of rSAG1/2 (Vet L-10) (*P* < 0.05, ANOVA). Spleen lymphocytes stimulated with Con A (1 μg/mL) from different groups were found to produce similar IFN-γ fold changes (*P* > 0.05, ANOVA). Therefore, significant production of IFN-γ could be readily elicited in mice by peritoneal immunization with a single dose of PLG-rSAG1/2 MPs.

**Table 1.** IFN-γ production of spleen lymphocyte cultures from immunized mice.

Group^a^	IFN-γ (fold change)^b^	
	TsoAg	Con A
PLG-rSAG1/2 MPs (single I.P. dose)	5.3 ± 1.6^c^	2.6 ± 0.7^e^
rSAG1/2 (Vet L-10) (single I.P. dose)	1.0 ± 0.4^d^	2.4 ± 0.3^e^
rSAG1/2 (Vet L-10) (two I.P. doses)	1.2 ± 0.2^d^	2.5 ± 0.4^e^
rSAG1/2 (single I.P. dose)	1.1 ± 0.3^d^	2.3 ± 0.6^e^
PLG (single I.P. dose)	1.0 ± 0.1^d^	2.1 ± 0.4^e^
PBS (single I.P. dose)	0.9 ± 0.2^d^	2.1 ± 0.2^e^

a Twelve weeks after immunization, spleen lymphocytes isolated from different groups of mice were stimulated with 5 μg/mL of TsoAg or 1 μg/mL of Con A for 6 h at 37 °C. Total cellular RNA was then extracted for the assay of IFN-γ production by real-time PCR.

b The fold change was determined by dividing the quantity of specific IFN-γ mRNA from stimulated cells by the quantity of IFN-γ mRNA from the unstimulated cells.

c–e A significant difference (*P* < 0.05) exists between groups with different superscript letters.

### Protection against *T. gondii* in mice induced by single immunization with PLG-rSAG1/2 MPs

**Figure 4.**
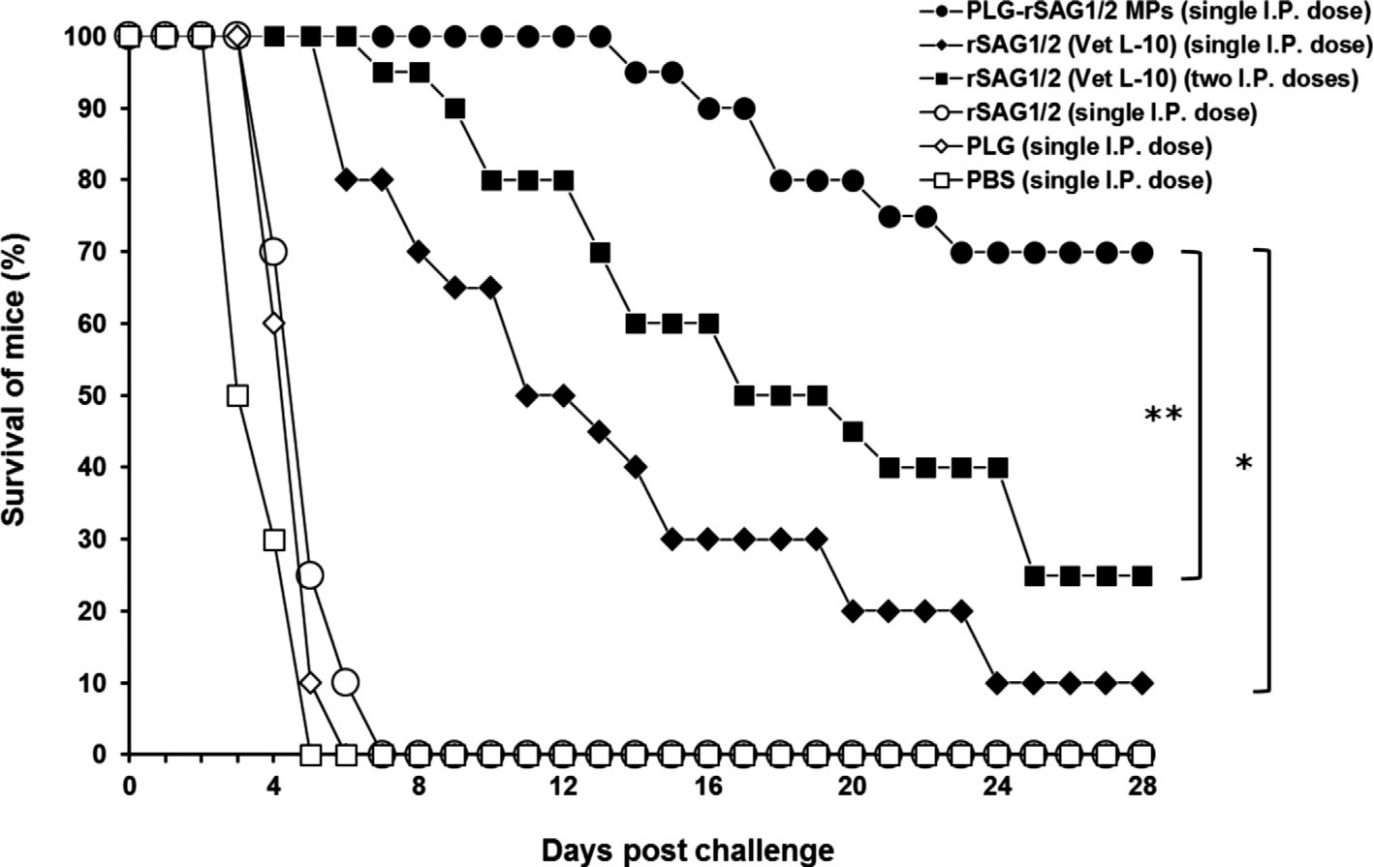
*Survival of immunized mice after a lethal tachyzoite challenge*. Groups of mice were peritoneally immunized with one dose of PLG-rSAG1/2 MPs (●), one dose of rSAG1/2 (Vet L-10) (◆), two doses of rSAG1/2 (Vet L-10) (■), one dose of rSAG1/2 alone (○), one dose of blank PLG (◇), or one dose of PBS (□). Twelve weeks after immunization, six groups of 20 mice each were subcutaneously infected with 1 × 10^4^ live tachyzoites of *T. gondii* (RH strain). Animals were observed daily for 28 days and the final survival rates were calculated. All groups were analyzed by the chi-square test. An asterisk (*) indicates *P <* 0.05 when comparing the PLG-rSAG1/2 (single I.P. dose) group to the rSAG1/2 (Vet L-10) (single I.P. dose) group. Two asterisks (**) indicate *P <* 0.05 when comparing the PLG-rSAG1/2 (single I.P. dose) group to the rSAG1/2 (Vet L-10) (two I.P. doses) group.

Twelve weeks after immunization, all groups of 20 mice each were subcutaneously challenged with 1 × 10^4^ live tachyzoites of *T. gondii* (RH strain). Mice were observed daily for an additional month (28 days) and the survival rates were recorded as shown in Figure 4. In the group of mice given a single peritoneal administration of PLG-rSAG1/2 MPs, six mice died between days 14 and 22 after challenge, showing a survival rate of 70%. A very low survival rate of 10% after challenge was detected in mice given one shot of rSAG1/2 (Vet L-10). Five out of 20 mice peritoneally immunized with two shots of rSAG1/2 (Vet L-10) survived, showing low protection of 25%. In contrast, single peritoneal immunization with rSAG1/2 alone, blank PLG, or PBS failed to generate protection against the tachyzoite infection in mice. Therefore, single peritoneal vaccination with rSAG1/2 encapsulated in PLG MPs elicited a significantly higher survival rate than peritoneal immunization with one or two shot(s) of rSAG1/2 (Vet L-10) (*P* < 0.05, chi-square test). These results indicate that the immunity induced by a single dose of PLG-rSAG1/2 MPs via the intraperitoneal route conferred solid resistance in mice to the experimental tachyzoite challenge.

## Discussion

Generally, purified proteins like rSAG1/2 are poorly immunogenic and therefore require effective adjuvants to aid them to elicit strong immunity [10]. In the present study, specific immune responses to both SAG1 and SAG2 proteins in TsoAg were elicited in mice by peritoneal immunization with rSAG1/2 protein formulated in either PLG microparticles or the Vet L-10 oil adjuvant (Fig. 1), indicating the importance of use of adjuvants. Therefore, rSAG1/2 protein could elicit both anti-SAG1 and anti-SAG2 immune responses in the mouse following one- or two-time immunization only when rSAG1/2 protein was initially formulated with adjuvants, but not in its soluble form.

The development of effective single-dose anti-*Toxoplasma* vaccines formulated with potent adjuvants, such as PLG, that are able to elicit long-lasting protective immunity, would be a critical strategy in the successful control of toxoplasmosis. In the present study, we found that single peritoneal immunization with PLG-rSAG1/2 MPs in mice resulted in not only long-lasting (12 weeks) triphasic IgG titers (Fig. 2) and lymphocyte proliferation (Fig. 3), but also efficacious protection (70%) against a lethal subcutaneous tachyzoite challenge (Fig. 4). However, peritoneal immunization with one or two shot(s) of rSAG1/2 formulated with the Vet L-10 adjuvant was unable to maintain protective immunity against *T. gondii* in mice (Figs. 2 and 3). Single immunization with PLG-rSAG1/2 MPs may therefore be better than one or two shot(s) of the oil formulation, rSAG1/2 (Vet L-10), in eliciting long-lasting anti-*Toxoplasma* immunity. We have demonstrated the feasibility of using PLG-rSAG1/2 MPs as a single-dose vaccine against *T. gondii* in mice.

The ability of PLG MPs to control the sustained release rate of entrapped antigens can induce long-lasting immunity in animals following single vaccination [9, 12]. Based on earlier studies, PLG MPs may allow pulsed and/or slow release of antigens to stimulate the immune system [22]. According to our previous *in vitro* release study in PBS, a long 56-day release process of rSAG1/2 protein from PLG MPs was divided into three distinct phases made up of an initial pulsed release (32.4% of total rSAG1/2 load) in the first three days, a very slow release (38% of total rSAG1/2 load) for the following 48 days, and a final pulsed release (18.1% of total rSAG1/2 load) during the last five days (days 52–56) [3]. In fact, previous studies have indicated that such triphasic controlled-release profiles result from the initial fast diffusion of protein absorbed onto the surface of PLG MPs, the very slow diffusion of encapsulated protein, and the final fast protein diffusion due to the bulk degradation of PLG MPs [17, 26, 28]. These release patterns are governed by the PLG copolymer degradation rate, which largely depends on the encapsulation conditions including the physical properties of the PLG polymer such as molecular weight, hydrophilicity, and the ratio of lactide to glycolide, as well as the MP features such as the size, morphology, and encapsulation efficiency [22].

Furthermore, the initial and final pulsed release in the triphasic release profile, respectively, resemble prime and boost immunization usually seen in a conventional vaccination process [28]. From this viewpoint, single immunization with PLG-rSAG1/2 MPs capable of performing the triphasic controlled release of rSAG1/2 protein can be thought of as administering two doses of rSAG1/2 protein. Therefore, PLG-rSAG1/2 MPs, administered in the mouse peritoneal cavity, gave rise to an initial pulsed release of rSAG1/2 to elicit early immunity induction (IgG titers and lymphocyte proliferation) in the first four weeks (Figs. 2 and 3). After clearance of the initial pulsed release of rSAG1/2, the very slow release of rSAG1/2 led to an insignificant increase in mouse immunity and, thus enabled the immunity to remain relatively constant from week 4 to week 8 (Figs. 2 and 3). Finally, the second induction in immunity from week 8 to week 12 (Figs. 2 and 3) could be accounted for as a result of the final pulsed release of rSAG1/2 protein due to the bulk degradation of PLG MPs during the period from day 52 to 56. In the present study, the triphasic fluctuation found in mouse IgG titers and lymphocyte proliferation indeed reflected the critical effect of triphasic rSAG1/2 release on *in vivo* anti-*Toxoplasma* immune responses. In addition, based on these results, we also believe that the *in vivo* release of rSAG1/2 protein in mice may be similar to the *in vitro* triphasic release in PBS observed in our previous study [3].

According to numerous crucial studies, vaccines based on PLG MPs are able to induce both humoral and cell-mediated immune responses in animals [10, 24]. An indicative hallmark of an efficacious *Toxoplasma* vaccine is the ability to induce strong Th1 cell-mediated immunity in animals [14]. In addition, IFN-γ, one of the Th1-type cytokines, has been demonstrated to be a decisive mediator of resistance to *T. gondii* [15, 20, 27]. Our previous studies have also shown that induction of both lymphocyte proliferation and IFN-γ production positively correlates with protective Th1 cell-mediated immunity against *T. gondii* [3–5]. In the present study, strong long-lasting lymphocyte proliferation was readily observed in mice that received a single dose of PLG-rSAG1/2 MPs (Fig. 3). Moreover, before challenge (12 weeks after immunization), mice given single peritoneal immunization with PLG-rSAG1/2 MPs produced significant amounts of IFN-γ (Table 1). Therefore, one dose of PLG-rSAG1/2 MPs could induce Th1 cell-mediated immunity required for prevention of *T. gondii* infection. On the other hand, anti-*Toxoplasma* IgG titers detected in the mouse serum following single immunization with PLG-rSAG1/2 MPs (Fig. 2) are consistent with those of previous studies, which have pointed out that humoral response should contribute to resistance against *T. gondii* [13, 16]. However, a further dye test would be necessary to measure the functional lytic activity of these antibodies. Therefore, one shot of PLG-rSAG1/2 MPs via the peritoneal route could induce mixed anti-*Toxoplasma* Th1/Th2 immune responses in mice. Although induction of IFN-γ is a decisive mechanism in the prevention of *T. gondii* infection, production of both IL-4 and IL-10 is still necessary to control lethal inflammation [1]. Detailed studies are therefore needed to assay these cytokine profiles. In addition, further measurements of memory T-cell markers (CD44 (low) CD62L (high) CD122 (high) sca-1 (+)) [18] would certainly be worth investigation to elucidate long-term immunity induced by PLG-rSAG1/2 MPs.

The long-lasting protective immunity induced by single immunization with PLG-rSAG1/2 MPs further protected 70% of experimentally challenged mice from lethal subcutaneous tachyzoite infection and allowed mice to survive for a long period of 28 days after the experimental challenge (Fig. 4). In further comparison, one dose of PLG-rSAG1/2 MPs elicited a significantly higher protective rate in mice than either one or two shot(s) of the oil formulation, rSAG1/2 (Vet L-10). Therefore, the notable vaccine potency indicates that the sustained release of rSAG1/2 protein from PLG MPs truly confers a substantial effect on the induction of protective anti-*Toxoplasma* immunity. PLG-rSAG1/2 MPs also show potential for being designed as a single-dose vaccine following further improvements such as enhancement of protein load in PLG MPs, as well as optimization and stabilization of protein release [32]. In addition, further studies using different parasite strains in large animals, especially livestock such as sheep and pigs, through different administration routes, are needed to corroborate the conclusions drawn from the mouse model.

In conclusion, we have extended our previous study in sustained release of rSAG1/2 protein to evaluate the possibility of the use of PLG-rSAG1/2 MPs as a single-dose vaccine against *T. gondii*. A single dose of PLG-rSAG1/2 MPs capable of sustaining rSAG1/2 release in a triphasic profile induces long-lasting triphasic immunity and strong protection against *T. gondii* in mice. Our study therefore provides notable evidence to indicate the feasibility of the development of a single-dose vaccine against toxoplasmosis based on PLG-rSAG1/2 MPs.

## Conflict of interest

The authors declare that they have no competing interests.

## References

[1] Bessieres MH, Swierczynski B, Cassaing S, Miedouge M, Olle P, Seguela JP, Pipy B. 1997 Role of IFN-gamma, TNF-alpha, IL4 and IL10 in the regulation of experimental *Toxoplasma gondii* infection. Journal of Eukaryotic Microbiology, 44(6), 87S. 9508466 10.1111/j.1550-7408.1997.tb05800.x

[2] Buxton D. 1993 Toxoplasmosis: the first commercial vaccine. Parasitology Today, 9(9), 335–337. 15463799 10.1016/0169-4758(93)90236-9

[3] Chuang SC, Ko JC, Chen CP, Du JT, Yang CD. 2013 Encapsulation of chimeric protein rSAG1/2 into poly(lactide-co-glycolide) microparticles induces long-term protective immunity against *Toxoplasma gondii* in mice. Experimental Parasitology, 134(4), 430–437. 23624036 10.1016/j.exppara.2013.04.002

[4] Chuang SC, Ko JC, Chen CP, Du JT, Yang CD. 2013 Induction of long-lasting protective immunity against *Toxoplasma gondii* in BALB/c mice by recombinant surface antigen 1 protein encapsulated in poly (lactide-co-glycolide) microparticles. Parasites & Vectors, 6, 34. 23398973 10.1186/1756-3305-6-34 PMC3584932

[5] Chuang SC, Yang CD. 2014 Sustained release of recombinant surface antigen 2 (rSAG2) from poly(lactide-co-glycolide) microparticles extends protective cell-mediated immunity against *Toxoplasma gondii* in mice. Parasitology, 141(12), 1657–1666. 10.1017/S0031182014000997 25036078

[6] Contini C. 2008 Clinical and diagnostic management of toxoplasmosis in the immunocompromised patient. Parassitologia, 50(1–2), 45–50. 18693556

[7] Delhaes L, Ajzenberg D, Sicot B, Bourgeot P, Darde ML, Dei-Cas E, Houfflin-Debarge V. 2010 Severe congenital toxoplasmosis due to a *Toxoplasma gondii* strain with an atypical genotype: case report and review. Prenatal Diagnosis, 30(9), 902–905. 20582922 10.1002/pd.2563

[8] Dubey JP. 2008 The history of *Toxoplasma gondii* – the first 100 years. Journal of Eukaryotic Microbiology, 55(6), 467–475. 19120791 10.1111/j.1550-7408.2008.00345.x

[9] Gupta RK, Singh M, O’Hagan DT. 1998 Poly(lactide-co-glycolide) microparticles for the development of single-dose controlled-release vaccines. Advanced Drug Delivery Reviews, 32(3), 225–246. 10837646

[10] Heegaard PM, Dedieu L, Johnson N, Le Potier MF, Mockey M, Mutinelli F, Vahlenkamp T, Vascellari M, Sorensen NS. 2011 Adjuvants and delivery systems in veterinary vaccinology: current state and future developments. Archives of Virology, 156(2), 183–202. 21170730 10.1007/s00705-010-0863-1

[11] Hill D, Dubey JP. 2002 *Toxoplasma gondii*: transmission, diagnosis and prevention. Clinical Microbiology and Infection, 8(10), 634–640. 12390281 10.1046/j.1469-0691.2002.00485.x

[12] Jain S, O’Hagan DT, Singh M. 2011 The long-term potential of biodegradable poly(lactide-co-glycolide) microparticles as the next-generation vaccine adjuvant. Expert Review of Vaccines, 10(12), 1731–1742. 22085176 10.1586/erv.11.126

[13] Johnson LL, Sayles PC. 2002 Deficient humoral responses underlie susceptibility to *Toxoplasma gondii* in CD4-deficient mice. Infection and Immunity, 70(1), 185–191. 11748181 10.1128/IAI.70.1.185-191.2002 PMC127596

[14] Jongert E, Roberts CW, Gargano N, Forster-Waldl E, Petersen E. 2009 Vaccines against *Toxoplasma gondii*: challenges and opportunities. Memórias do Instituto Oswaldo Cruz, 104(2), 252–266. 10.1590/s0074-02762009000200019 19430651

[15] Jongert E, Lemiere A, Van Ginderachter J, De Craeye S, Huygen K, D’Souza S. 2010 Functional characterization of *in vivo* effector CD4(+) and CD8(+) T cell responses in acute Toxoplasmosis: an interplay of IFN-gamma and cytolytic T cells. Vaccine, 28(13), 2556–2564. 20117266 10.1016/j.vaccine.2010.01.031

[16] Kang H, Remington JS, Suzuki Y. 2000 Decreased resistance of B cell-deficient mice to infection with *Toxoplasma gondii* despite unimpaired expression of IFN-gamma, TNF-alpha, and inducible nitric oxide synthase. Journal of Immunology, 164(5), 2629–2634. 10.4049/jimmunol.164.5.2629 10679102

[17] Kavanagh OV, Earley B, Murray M, Foster CJ, Adair BM. 2003 Antigen-specific IgA and IgG responses in calves inoculated intranasally with ovalbumin encapsulated in poly(DL-lactide-co-glycolide) microspheres. Vaccine, 21(27–30), 4472–4480. 14505930 10.1016/s0264-410x(03)00432-8

[18] Klebanoff CA, Scott CD, Leonardi AJ, Yamamoto TN, Cruz AC, Ouyang C, Ramaswamy M, Roychoudhuri R, Ji Y, Eil RL, Sukumar M, Crompton JG, Palmer DC, Borman ZA, Clever D, Thomas SK, Patel S, Yu Z, Muranski P, Liu H, Wang E, Marincola FM, Gros A, Gattinoni L, Rosenberg SA, Siegel RM, Restifo NP. 2016 Memory T cell-driven differentiation of naive cells impairs adoptive immunotherapy. Journal of Clinical Investigation, 126(1), 318–334. 26657860 10.1172/JCI81217 PMC4701537

[19] Liu Q, Singla LD, Zhou H. 2012 Vaccines against *Toxoplasma gondii*: status, challenges and future directions. Human Vaccines & Immunotherapeutics, 8(9), 1305–1308. 22906945 10.4161/hv.21006 PMC3579912

[20] Mammari N, Vignoles P, Halabi MA, Dardé ML, Courtioux B. 2015 Interferon gamma effect on immune mediator production in human nerve cells infected by two strains of *Toxoplasma gondii*. Parasite, 22, 39. 26692261 10.1051/parasite/2015039 PMC4686326

[21] Overbergh L, Valckx D, Waer M, Mathieu C. 1999 Quantification of murine cytokine mRNAs using real time quantitative reverse transcriptase PCR. Cytokine, 11(4), 305–312. 10328870 10.1006/cyto.1998.0426

[22] Raman C, Berkland C, Kim K, Pack DW. 2005 Modeling small-molecule release from PLG microspheres: effects of polymer degradation and nonuniform drug distribution. Journal of Controlled Release, 103(1), 149–158. 15773062 10.1016/j.jconrel.2004.11.012

[23] Robert-Gangneux F, Darde ML. 2012 Epidemiology of and diagnostic strategies for toxoplasmosis. Clinical Microbiology Reviews, 25(2), 264–296. 22491772 10.1128/CMR.05013-11 PMC3346298

[24] Sivakumar SM, Safhi MM, Kannadasan M, Sukumaran N. 2011 Vaccine adjuvants – current status and prospects on controlled release adjuvancity. Saudi Pharmaceutical Journal, 19(4), 197–206. 23960760 10.1016/j.jsps.2011.06.003 PMC3744968

[25] Sousa S, Canada N, Correia da Costa JM, Darde ML. 2010 Serotyping of naturally *Toxoplasma gondii* infected meat-producing animals. Veterinary Parasitology, 169(1–2), 24–28. 20083355 10.1016/j.vetpar.2009.12.025

[26] Sturesson C, Carlfors J. 2000 Incorporation of protein in PLG-microspheres with retention of bioactivity. Journal of Controlled Release, 67(2–3), 171–178. 10825551 10.1016/s0168-3659(00)00205-4

[27] Suzuki Y, Orellana MA, Schreiber RD, Remington JS. 1988 Interferon-gamma: the major mediator of resistance against *Toxoplasma gondii*. Science, 240(4851), 516–518. 3128869 10.1126/science.3128869

[28] Uchida M, Natsume H, Kishino T, Seki T, Ogihara M, Juni K, Kimura M, Morimoto Y. 2006 Immunization by particle bombardment of antigen-loaded poly-(DL-lactide-co-glycolide) microspheres in mice. Vaccine, 24(12), 2120–2130. 16356602 10.1016/j.vaccine.2005.11.027

[29] Verma R, Khanna P. 2013 Development of *Toxoplasma gondii* vaccine: a global challenge. Human Vaccines & Immunotherapeutics, 9(2), 291–293. 23111123 10.4161/hv.22474 PMC3859749

[30] Weiss LM, Dubey JP. 2009 Toxoplasmosis: a history of clinical observations. International Journal for Parasitology, 39(8), 895–901. 19217908 10.1016/j.ijpara.2009.02.004 PMC2704023

[31] Yang CD, Chang GN, Chao D. 2004 Protective immunity against *Toxoplasma gondii* in mice induced by a chimeric protein rSAG1/2. Parasitology Research, 92(1), 58–64. 14605877 10.1007/s00436-003-0992-5

[32] Ye M, Kim S, Park K. 2010 Issues in long-term protein delivery using biodegradable microparticles. Journal of Controlled Release, 146(2), 241–260. 20493221 10.1016/j.jconrel.2010.05.011

[33] Zhang NZ, Chen J, Wang M, Petersen E, Zhu XQ. 2013 Vaccines against *Toxoplasma gondii*: new developments and perspectives. Expert Review of Vaccines, 12(11), 1287–1299. 24093877 10.1586/14760584.2013.844652

